# A study on the correlation between the perception of intelligent college English learning environments and the willingness to communicate in listening, speaking, reading, and writing

**DOI:** 10.3389/fpsyg.2026.1885211

**Published:** 2026-07-08

**Authors:** Yan Li

**Affiliations:** School of Foreign Languages, Lanzhou City University, Lanzhou, China

**Keywords:** foreign language learning anxiety, generative AI, intelligent learning environment, L2 self-efficacy, multimodal willingness to communicate

## Abstract

**Introduction:**

As generative AI tools become integrated into educational settings, foreign language teaching and learning are gradually adapting. This cross-sectional mixed-methods study examines the relationships between college students’ perceptions of intelligent learning environments and their multimodal L2 willingness to communicate (WTC), while analyzing L2 self-efficacy as a potential mediator and foreign language anxiety (FLA) as a moderator.

**Methods:**

Survey and qualitative data were collected from 960 college students. Quantitative analyses were conducted to examine the associations between perceptions of intelligent environments, L2 self-efficacy, FLA, and WTC across both receptive (listening, reading) and productive (speaking, writing) modalities. Qualitative feedback was analyzed to contextualize these relationships.

**Results:**

The survey data indicated a significant positive association between the perception of intelligent environments and overall WTC. Although baseline WTC for productive skills was lower than for receptive tasks, favorable perceptions of AI showed comparable positive associations with communication willingness across both modalities. L2 self-efficacy served as an indirect link, accounting for 56.19% of the total association. Exploratory analyses indicated that high self-efficacy exhibited a potential buffering tendency against the negative correlation between FLA and WTC, though this interaction did not reach statistical significance (*p* = 0.068). Multi-group analyses demonstrated that these positive associations were consistent across different language proficiency levels. Qualitatively, AI platforms were characterized as perceived “psychological safety buffers” with lower social-evaluative risks.

**Discussion:**

These cross-sectional insights suggest that favorable perceptions of intelligent environments are positively related to L2 communication willingness. Rather than treating these environments merely as technical aids, educators might leverage their low-pressure attributes as supportive digital scaffolding while monitoring cognitive dependence to encourage eventual autonomous communication.

## 1 Introduction

As generative AI tools become increasingly integrated into educational settings, college students’ foreign language cognition and acquisition processes are experiencing a transition from mere knowledge acquisition to “human-machine collaborative” interaction (Alshamy et al., 2025; Klimova et al., 2024; Lo et al., 2024). Rather than serving merely as auxiliary aids for vocabulary retrieval or grammar correction, these algorithms now act as intelligent agents. Equipped with natural language capabilities, they frequently participate in learners’ cognitive processing and emotional regulation processes (Slamet, 2024; Wang and Fan, 2025; Yu et al., 2024). This continuous technological adaptation raises a relevant empirical question: when algorithms provide immediate cognitive scaffolding and bear part of the language processing load, does learners’ Willingness to Communicate (WTC) in an L2 environment exhibit an observable correlation with these tools?

L2 willingness to communicate (WTC) is conventionally regarded as a core predictor of actual language use behavior. Based on this premise, the classic heuristic model of L2 WTC (MacIntyre et al., 1998) reveals a dynamic mechanism ranging from stable personality traits to immediate situational impulses (Chen et al., 2021; Cheng and Xu, 2022; Ma et al., 2023). Building on this, prior studies also show that teaching formats with lower pressure and stronger interactivity, such as gamified learning, are positively associated with EFL learners’ WTC, indicating that WTC has strong situational sensitivity to the external learning environment (Liu et al., 2021). Recent scholarship further highlights that learners’ WTC is highly dynamic and particularly sensitive to modern digital contexts. For instance, AI-driven informal digital learning environments demonstrate significant potential to shape learners’ L2 speaking motivation and WTC by providing flexible and personalized inputs (Solhi et al., 2026). Yet much of the psychological research on WTC has traditionally focused on interpersonal interactions marked by physical presence, leaving the exploration of communicative psychology in digital environments, especially intelligent tool environments, remains insufficient. Unlike traditional face-to-face communication, generative AI tools provide learners with an interactive situation of “de-socialized evaluation.” Because this interface strips away much of the incidental face threat and peer pressure found in regular classrooms, it is worth asking how exactly students’ perceptual experiences of this intelligent environment relate to their communicative tendencies in different pragmatic modalities (Deng et al., 2024; Yan et al., 2025).

However, assessing learners’ acceptance of intelligent environments requires acknowledging a “dark side.” Emerging studies applying the Technology Acceptance Model (TAM) indicate that while generative AI functions as cognitive scaffolding, excessive reliance can trigger digital fatigue and AI dependency (Honggang et al., 2026; Khoso et al., 2025b). Prolonged technological dependency may exacerbate learner burnout and hinder the achievement of Sustainable Development Goals (SDG-4) in EFL classrooms (Khoso et al., 2026). The dual forces of technological support and cognitive dependency must therefore be integrated when analyzing students’ psychological state during AI-mediated communication.

Translating external technological perceptions into internal behavioral willingness is a complex process, likely involving cognitive and emotional filters. From a social cognitive perspective, a smooth user experience may correspond to a learner’s positive assessment of their language task competence—namely, L2 self-efficacy—suggesting a potential indirect link for this variable. Alongside cognitive factors, the precise role of an emotional variable like Foreign Language Anxiety (FLA) in the algorithmic era also warrants re-examination (Chong et al., 2025; Deepika and Jennifer, 2025; Elov et al., 2025). When the predictable feedback of human-machine collaboration coexists with the uncertain risks of actual interpersonal communication, does high anxiety still show a strong negative correlation with the link between efficacy and willingness?

Broadening the scope further, technological associations might also vary across specific language skills. For receptive dimensions like listening and reading, tool assistance mainly acts on information decoding, meaning individuals face relatively low social exposure risks. Conversely, productive tasks—speaking and writing—require learners to construct self-identity and formulate deeper expressions, naturally involving more complex psychological defense mechanisms. Given these differences, treating generative AI merely as a unified scaffold is insufficient; researchers must distinguish its function as an interlocutor for productive practice from its role as a tool for receptive task support (Khoso et al., 2025a). Clarifying these psychological differences based on modality characteristics helps to more accurately evaluate how students interact with intelligent foreign language learning systems.

Motivated by these theoretical extensions and realistic contexts, this study aims to examine the following progressive research questions through a cross-sectional survey and qualitative feature analysis targeting the college student population, seeking to outline the associative patterns of learners’ communicative psychology under this technological trend:

Question 1: Is there a significant positive correlation between college students’ perceptual experience of intelligent English learning environments and their multimodal (listening, speaking, reading, and writing) willingness to communicate?

Question 2: (RQ2a) Given the varying social risks across communicative modalities, does willingness to communicate present an asymmetrical distribution in different pragmatic dimensions?

(RQ2b) And does the predictive strength of AI perception differ when comparing receptive willingness (listening/reading) with productive willingness (speaking/writing)?

Question 3: Does domain-specific L2 self-efficacy play a significant mediating role in the associative path between environmental perception and willingness to communicate?

Question 4: In an intelligent environment, does foreign language learning anxiety act as a negative moderating variable, restricting the associative strength between self-efficacy and willingness to communicate? Is there an observable “buffering pattern” associated with technical assistance?

Question 5: Does the aforementioned multi-dimensional psychological mediation model possess cross-group stability among subsamples categorized by different baseline English levels?

## 2 Literature review and theoretical model construction

### 2.1 AI-assisted learning environments and psychological regulation of cognitive load

As generative AI gains traction, the landscape of foreign language acquisition is gradually adapting to these new technological affordances (Alshammari, 2025; Heydarnejad, 2025). Drawing on the technology acceptance model (TAM), learners’ adoption of information systems extends beyond objective technical parameters to rely heavily on “perceived usefulness” and “perceived ease of use” Translated into L2 learning scenarios, the psychological relevance of these perceptions likely involves the regulation of cognitive load. When confronted with demanding cross-lingual analysis or text reconstruction tasks, beginners might experience working memory overload; rather than reaching psychological exhaustion, they can increasingly rely on AI tools as external cognitive scaffolds that offer contextualized instant translation, syntactic deconstruction, and logical error correction. Once learners recognize this low-threshold technical support as a reliable “digital backup resource,” their defensive withdrawal from complex language tasks might soften, a shift that shares a theoretical correlation with the subsequent generation of communicative intentions (Derakhshan and Lalli, 2025; Han, 2025).

### 2.2 Theoretical expansion of multimodal L2 WTC and face threats

Following the layered heuristic (3D) model of WTC proposed by MacIntyre et al. (1998), communicative willingness is broadly understood neither strictly as a fixed trait nor a pure emotion, but rather a multi-dimensional and transient state of readiness dynamically tied to situational variables. Prior findings demonstrate positive correlations between WTC and specific teaching conditions—gamified learning, classroom interactions, and teacher support—affirming that this readiness remains highly sensitive to its immediate learning environment (Liu et al., 2021; Hu and Wang, 2023). Recognizing the highly mediated nature of AI contexts, it becomes necessary to segment traditional WTC measurements by modality. Social evaluation anxiety often accompanies communicative attempts, creating distinct psychological boundary lines between “receptive willingness” (listening and reading) and “productive willingness” (speaking and writing) (Hu and Wang, 2023; Jelínková et al., 2023). Because listening and reading operate at the input stage, learners can use AI for meaning construction from a bystander’s perspective, In this context, generative AI predominantly functions as a cognitive tool for receptive task support (e.g., summarization, translation), virtually eliminating risks to their social image. Speaking and writing, requiring active recoding and self-disclosure, inherently carry much higher face-threat attributes. Here, AI typically acts as an interlocutor for productive practice (e.g., conversation simulation) (Khoso et al., 2025a). Assessing the relative predictive power of intelligent perception across these distinct modalities offers a clearer view of how technological support relates to students’ communicative participation (Lee and Taylor, 2024).

### 2.3 Internalization and the mediating mechanism of L2 self-efficacy

Bridging the conceptual gap between technological perception and behavioral willingness, Bandura’s self-efficacy theory supplies a helpful explanatory framework. Defined as an individual’s belief in their domain-specific competence, efficacy might be nurtured during intelligent human-machine collaboration, where continuous and accurate AI feedback generates high-frequency, low-risk “vicarious success experiences” (Celik et al., 2025; Guo et al., 2023). What remains to be tested in cross-sectional models is whether this technological linkage undergoes a true internalization process—that is, whether learners psychologically convert “task completion with AI” into an affirmation of their own language processing capacity. Should external perceptions map onto domain-specific self-efficacy, this internal conviction could plausibly manifest as an increased readiness to approach real interpersonal communicative situations (Liu et al., 2025).

### 2.4 The buffering and moderating effects of foreign language learning anxiety

Widely recognized as a restrictive emotional variable in language acquisition, Foreign Language Anxiety (FLA) consistently shows a negative correlation with WTC in the post-stages spanning from ability to practice. Recent holistic perspectives have further confirmed that negative emotional orientations, encompassing both state anxiety and boredom, can severely inhibit EFL learners’ WTC across both in-person and digital learning contexts (Enferad et al., 2025; Solhi, 2024). Yet the inherent privacy of modern human-computer interaction prompts a re-evaluation of its boundary conditions. Operating without the moral evaluations or scrutinizing gaze of human interlocutors, an emotionless AI entity inherently lacks the core triggers for FLA typically found in traditional classrooms, specifically the “fear of negative evaluation” (Lee et al., 2024; Yan et al., 2025). Moving beyond peer pressure, traditional cross-cultural communication involves extensive ambiguity, where successful interactions rely heavily on a learner’s intrinsic tolerance for uncertainty (Huang and Zhou, 2025). AI applications, utilizing powerful context generation and parsing capabilities, objectively filter much of this cognitive hindrance, theoretically easing the anxiety linked to cross-cultural ambiguity. Evaluating whether deeply rooted trait anxiety might be buffered in tech-supported scenarios, or if it maintains a stark negative correlation with the shift from efficacy to willingness, adds valuable nuance to the psychological profile of digital-age learners. At the same time, this psychological profile must account for the counter-narrative of technological disruption. As learners rely heavily on localized AI scaffolding to manage their learning tasks, they may develop a psychological dependence on these tools, which could subsequently trigger apprehension when they are required to engage in linguistic performance without technological support (Khoso et al., 2025c). Consequently, evaluating the moderating role of FLA in contemporary contexts requires examining whether heightened self-efficacy can effectively navigate the traditional fears of negative human evaluation while simultaneously resisting the emerging potential anxieties induced by AI dependency.

### 2.5 Proposal of research hypotheses

Integrating these theoretical dimensions into a comprehensive structural model, this study outlines several hypotheses regarding main associations, structural heterogeneity, indirect links, and moderation patterns:

*H1* (main association): AI intelligent environment perception shows a significant positive correlation with college students’ multidimensional L2 willingness to communicate.

*H2* (modality heterogeneity): H2 is divided into two sub-hypotheses based on the distinct risks of pragmatic dimensions.

*H2a*: L2 communicative willingness presents a baseline asymmetrical distribution, where receptive WTC (listening/reading) is significantly higher than productive WTC (speaking/writing).

*H2b*: The positive predictive link between AI perception and “receptive willingness” will be significantly stronger than its association with “productive willingness.”

*H3* (self-efficacy mediation): Domain-specific self-efficacy plays a significant mediating role in the “AI perception → multimodal WTC” path.

*H4* (emotional moderation): Foreign language learning anxiety plays a negative moderating role in the “self-efficacy → WTC” stage; although within an intelligent context, this restrictive pattern may appear relatively weakened.

*H5* (group heterogeneity): The positive correlation paths among AI perception, self-efficacy, and WTC remain consistently significant across subsamples characterized by different baseline English proficiency levels, demonstrating cross-group stability.

**Figure 1:**
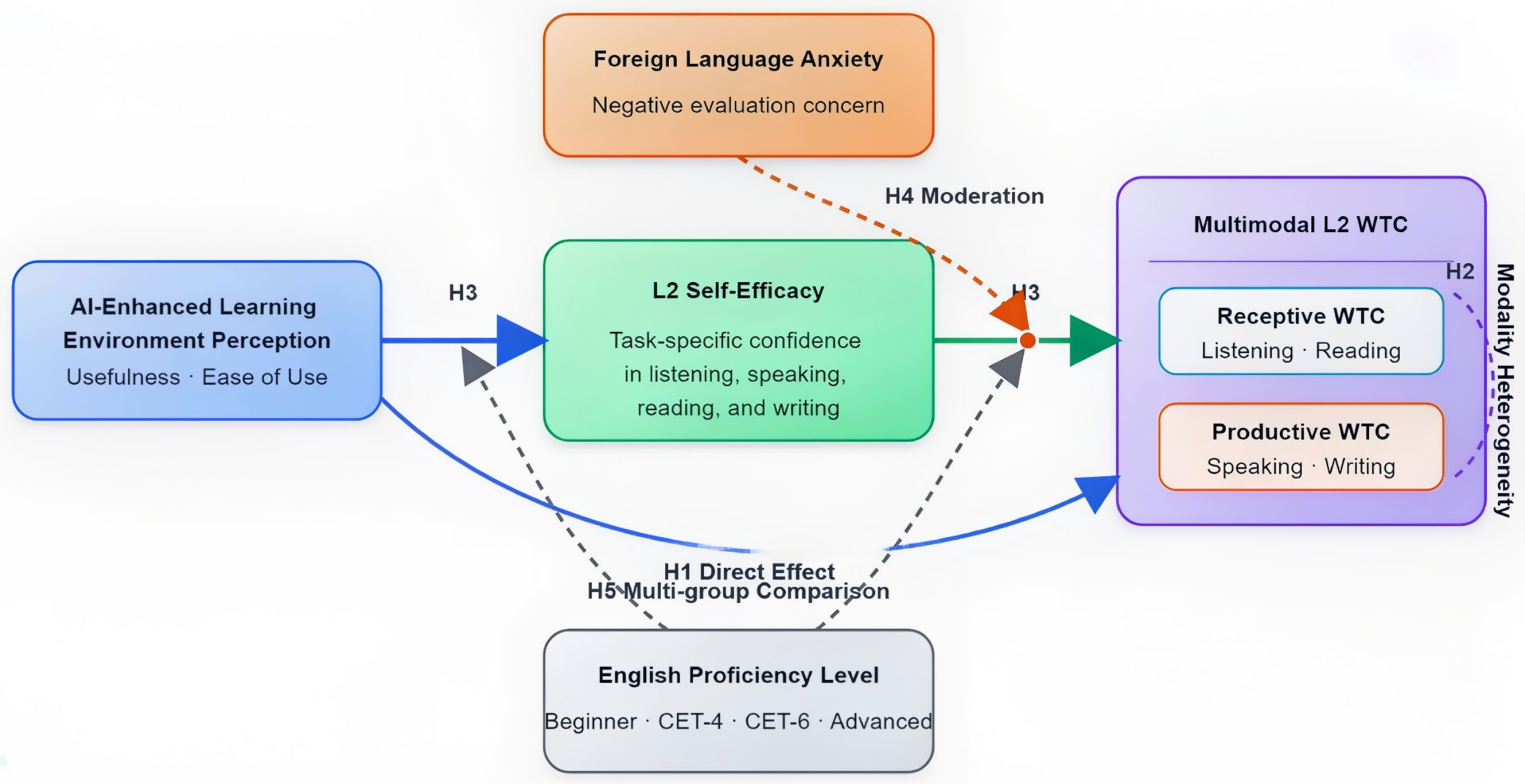
Conceptual model of intelligent learning environment perception, self-efficacy, foreign language anxiety, and multimodal L2 willingness to communicate. Solid arrows indicate hypothesized direct or mediating effects; dashed arrows indicate the moderating effect of foreign language learning anxiety and multi-group comparisons among different English proficiency groups. WTC, willingness to communicate; L2, second language.

Synthesizing these interconnected hypotheses, this study builds a conceptual model integrating intelligent learning environment perception, L2 self-efficacy, foreign language anxiety, and multimodal L2 willingness to communicate, as shown in Figure 1.

## 3 Research methods

### 3.1 Research design, data collection, and participant characteristics

This study adopted a cross-sectional mixed-methods design combining a quantitative survey with qualitative thematic analysis. The quantitative component was used to examine the structural associations among AI perception, self-efficacy, foreign language anxiety, and multimodal L2 WTC, whereas the qualitative component was used to provide supplementary learner narratives for interpreting the statistical patterns.

Data collection was concentrated between January and March 2026, primarily implemented through Wenjuanxing, a widely used online survey platform in China. The research team conducted targeted distributions relying on online learning communities and physical campus forums. All subjects entered the answering interface after voluntarily clicking the participation link. The survey collected an initial 1,030 questionnaires. After strictly excluding samples with abnormal response times (e.g., below routine reading response thresholds), obvious regular preferences (e.g., highly consistent choices), and messy or invalid qualitative feedback, 960 high-quality valid samples were finally retained (effective recovery rate 93.2%).

The sample characteristics show diverse distributions as seen in Table 1: females accounted for 59.6% and males 40.4%; major compositions covered STEM (25.6%), Economics/Management (25.8%), Liberal Arts/Education (30.1%), and Arts (fine arts, performing arts, and physical education; 18.4%), enhancing sample diversity in disciplinary backgrounds and avoiding conceptual overlap with the humanities. The English proficiency levels, combining self-assessment and objective tests, presented a clear stepped distribution: beginners who have not yet passed grade exams accounted for 15.0%, the main CET-4 group 43.0%, the advanced CET-6 group 31.6%, and learners with advanced certification capabilities 10.4%.

**Table 1:** Demographic characteristics distribution of subjects (*N* = 960).

Characteristic dimension	Category	Frequency (*n*)	Percentage (%)
Gender	Male	388	40.4%
	Female	572	59.6%
Grade	Freshman	273	28.4%
	Sophomore	302	31.5%
	Junior	199	20.7%
	Senior	186	19.4%
Major	STEM	246	25.6%
	Economics and management	248	25.8%
	Liberal arts and education	289	30.1%
	Arts	177	18.4%
English level	Beginner (below CET-4)	144	15.0%
	Intermediate (CET-4)	413	43.0%
	Advanced (CET-6)	303	31.6%
	High-level (professional cert.)	100	10.4%

Arts refers to fine arts, performing arts, and physical education, and is distinguished from liberal arts/education.

### 3.2 Measurement attributes and localized application of measurement tools

The measurement tools adopted in this study are all based on internationally classic psychological test scales. To ensure linguistic precision and conceptual equivalence in the targeted AI-assisted learning context, these scales underwent a rigorous translation and back-translation procedure. Initially, two applied linguistics professors independently translated the original English items into Chinese. Subsequently, a third bilingual expert back-translated the Chinese draft into English. Prior to the large-scale administration, a pilot test was conducted with 45 undergraduate EFL students to evaluate instructional clarity, readability, and cultural appropriateness. Based on their feedback, minor terminological refinements were made. All items are scored on a 5-point Likert scale. Detailed bilingual questionnaire items (English and localized Chinese versions) and the corresponding codebooks are available in Supplementary materials S1 and S2.

#### 3.2.1 AI intelligent perception scale

For the measurement of AI intelligent perception, referencing Davis’s (1989) Technology Acceptance Model (TAM) and Mac Callum and Jeffrey’s (2013) exploration of digital skill adoption behavior, we compiled the “AI Intelligent Tool Usage Experience and Perception Scale” containing 5 items. This scale focuses on learners’ intuitive experiences in dimensions such as “alleviating cognitive burden,” “immediacy of feedback,” “pleasure of efficiency,” and “technological path dependence.” All test items are scored via a 5-point Likert scale (1 = *strongly disagree*, 5 = *strongly agree*). For example, the item “When encountering difficulties in English learning, I tend to prioritize asking AI tools rather than skipping directly.”

#### 3.2.2 Domain-specific L2 self-efficacy scale

To accurately align with the concept of self-efficacy proposed by Bandura (1997) and adapt it to the context of Chinese foreign language learners, we adopted the domain-specific efficacy scale framework developed by Wang et al. (2014). This section contains 4 core measurement items, covering competence belief measurements in the four basic skills of listening, speaking, reading, and writing. The item design breaks through generic “I have confidence” statements and turns to specific task situations, such as capturing fast-speech information or writing organized short essays. This “specificity” ensures the explanatory power of the efficacy variable when dealing with multimodal communication.

#### 3.2.3 Foreign language anxiety perception scale (FLA)

Measurements of the emotional dimension primarily rely on the theoretical foundation of foreign language classroom anxiety established by Horwitz et al. (1986). Through simplification and situational adjustment, this section focuses via 4 measurement items on “peer pressure,” “impromptu response panic,” “fear of negative evaluation,” and “assessment anxiety.” This is not only a portrayal of learners’ current emotional states but also a means to observe the constraints these psychological “undercurrents” place on the transformation of willingness.

#### 3.2.4 Multimodal L2 willingness to communicate scale (L2 WTC)

As the key dependent variable of this study, the measurement of WTC referred to concepts from MacIntyre et al. (1998) and Peng and Woodrow (2010). To reflect the characteristics of the digital era, we disassembled WTC into two dimensions: “receptive willingness (listening and reading)” and “productive willingness (speaking and writing),” totaling 8 measurement items. This split has profound practical significance, because it covers a broad psychological spectrum from passively watching a non-subtitled video to actively publishing extensive English posts on social platforms, thereby acutely detecting the potential psychological tensions among different pragmatic modalities.

### 3.3 Expansion of the qualitative dimension and thematic coding

To compensate for the limitations of quantitative data in revealing deep individual emotions, this study appended an optional qualitative question (Part 3) at the end of the survey. The prompt specifically asked: “When using generative AI tools (such as ChatGPT or translation applications) for English learning, what specific benefits, cognitive shifts, or psychological anxieties have you experienced? Please describe your true feelings.” Due to the voluntary nature of this section, valid qualitative responses were obtained from 712 participants (excluding blank or meaningless inputs), with text lengths ranging from 15 to 120 words (averaging approximately 45 words).

Referring to Braun and Clarke (2006) thematic analysis method, two independent researchers used qualitative analysis software to conduct semantic segmentation, high-frequency word condensation (such as “no longer afraid of ridicule,” “sense of emptiness from reliance on machine translation”), and categorization on the responses. To ensure the trustworthiness and credibility of the qualitative coding (Nowell et al., 2017), the two coders initially reviewed 100 random responses to establish a unified codebook, achieving high inter-rater reliability (Cohen’s Kappa = 0.86). Any subsequent analytical disagreements were resolved through consensus discussions with a third researcher. As an important part of Triangulation, qualitative coding aims to assign concrete narrative explanations close to learners’ native experiences to the abstract paths provided by structural equations.

### 3.4 Data analysis methods

This study employs a mixed analysis strategy primarily quantitative with qualitative support. Quantitative data preprocessing and descriptive statistics were completed using IBM SPSS 26.0 software. Subsequently, AMOS 24.0 software was used to construct a Structural Equation Modeling (SEM) to examine the complex correlations among psychological latent variables. The specific quantitative analysis followed standard procedures: Firstly, referencing Podsakoff et al. (2003), Harman’s Single-Factor Test was used to screen for potential common method biases in the self-reported data. While acknowledging that this single-factor approach is widely considered a low-sensitivity diagnostic (Podsakoff et al., 2012), we used it only as an initial conservative baseline for data quality, and subsequent interpretations of correlational results carefully considered possible method effects. Secondly, Confirmatory Factor Analysis (CFA) was utilized to measure the convergent validity (AVE, CR) and discriminant validity (Fornell-Larcker criterion) of the measurement model, and comprehensively evaluate the model’s overall goodness of fit (such as *χ*^2^/*df*, RMSEA, CFI, etc.); Following the guidelines of Byrne (2016), model modifications were considered strictly based on Modification Indices (MI > 10.0) and theoretically justifiable semantic overlaps within the same latent construct, ensuring model parsimony and avoiding over-fitting; thirdly, Maximum Likelihood estimation was applied to test the main effects and predictive power of path coefficients between latent variables; subsequently, the Bootstrap test method based on 5,000 resamplings was adopted to accurately evaluate the confidence intervals of the mediation effect of self-efficacy; finally, Latent Interaction Modeling within the SEM framework (using the product-indicator approach) was applied to test the moderating effect of foreign language anxiety, and Multi-group Analysis was utilized to conduct exploratory subgroup path comparisons. For qualitative text data, two independent researchers used NVivo qualitative analysis software to conduct semantic segmentation, high-frequency word condensation, and thematic categorization coding on the 712 valid open-ended responses, offering deep narrative explanations close to learners’ native experiences for the abstract SEM statistical results in a triangulated manner.

## 4 Research results

### 4.1 Preliminary tests: common method bias and reliability and validity verification

Before conducting hypothesis testing and path analysis, this study first evaluated the reliability and construct validity of the self-reported data.

As a preliminary diagnostic check for common method bias (CMB), all measurement items were examined using Harman’s Single-Factor Test. The first unrotated factor explained 24.05% of the total variance, which was below the commonly used 40% reference threshold (Podsakoff et al., 2003), suggesting that no dominant single-factor structure was detected in the self-reported data. However, given the low sensitivity of this diagnostic approach, this result should not be interpreted as sufficient evidence to rule out CMB. Therefore, subsequent interpretations of correlation and path coefficients should remain cautious.

To assess the stability and convergent validity of the measurement tools, this study calculated related indices as shown in Table 2. The Cronbach’s *α* coefficients of all latent variables ranged between 0.702 and 0.780, and the Composite Reliability (CR) values were all greater than 0.8 (0.817–0.850), showing good internal consistency. Furthermore, the Average Variance Extracted (AVE) values for all latent variables ranged from 0.528 to 0.573, entirely exceeding the 0.5 threshold suggested by Fornell and Larcker (1981), indicating good convergent validity of the measurement model. Through Confirmatory Factor Analysis (CFA) testing, it was found that after establishing residual correlations within a few theoretically permissible bounds, the structural model exhibited good goodness of fit: *χ*^2^ = 338.55, *df* = 183, *χ*^2^/*df* = 1.850, CFI = 0.968, TLI = 0.962, RMSEA = 0.030, GFI = 0.958, SRMR = 0.043. All fit indices met the general standards suggested by Bentler (1990), indicating that the model fits well with its actual sample data. To avoid capitalizing on chance characteristics of the sample, post-hoc model modifications were conducted strictly within items of the same latent construct with Modification Indices (MI) greater than 10.0 (Kline, 2023). Specifically, three intra-factor residual pairs were correlated based on solid conceptual commonalities: e1 ↔ e2 (MI = 22.45) due to semantic overlap regarding technological efficiency; e8 ↔ e9 (MI = 18.12) based on modality homogeneity within productive speaking/writing tasks; and e15 ↔ e16 (MI = 14.33), reflecting contextual similarity in receptive decoding. These theoretical justifications ensure model parsimony and robustness while preventing over-fitting (Byrne, 2016). Detailed modification indices and theoretical rationales are provided in Supplementary material S3.

**Table 2:** Construct reliability and convergent validity analysis report.

Measurement construct	No. of items	Standardized CFA factor loadings	Cronbach’s *α*	Composite reliability (CR)	Average variance extracted (AVE)
AI intelligent perception (AI)	5	0.673–0.767	0.780	0.850	0.531
Self-efficacy (SE)	4	0.710–0.754	0.726	0.830	0.549
Foreign language anxiety (FLA)	4	0.698–0.761	0.702	0.817	0.528
Receptive WTC (WTC-R)	4	0.737–0.782	0.751	0.843	0.573
Productive WTC (WTC-P)	4	0.721–0.762	0.733	0.833	0.555

### 4.2 Descriptive statistics and discriminant validity of core variables

An initial examination of the correlation matrix highlights the foundational relational patterns among the main variables. Specifically, AI intelligent perception (*M* = 3.57) demonstrates significant positive correlations with students’ receptive and productive WTC (*r* = 0.220 and 0.224, *p* < 0.01), providing initial statistical support for the primary association proposed in Hypothesis H1. Alongside this technological perception, L2 self-efficacy shares robust positive links with these dual communication modalities (*r* = 0.414 and 0.375) while foreign language anxiety displays expected negative correlations (*r* = −0.200 and −0.285). To rigorously test the discriminant degree among constructs, this study introduced the Fornell-Larcker criterion as shown in Table 3. Results showed that the square root of the AVE for each construct (listed on the diagonal) was significantly greater than the correlation coefficients between that construct and other latent variables. Therefore, all core variables maintained clear structural boundaries at the psychometric level.

**Table 3:** Correlation matrix and discriminant validity test (Fornell-Larcker).

Variables	*M*	*SD*	1	2	3	4	5
1. AI perception	3.57	0.70	**0.729**				
2. Self-efficacy	3.38	0.70	0.346**	**0.741**			
3. FL anxiety	3.02	0.70	−0.155**	−0.224**	**0.727**		
4. Receptive WTC	3.66	0.71	0.220**	0.414**	−0.200**	**0.757**	
5. Productive WTC	3.10	0.72	0.224**	0.375**	−0.285**	0.482**	**0.745**

Numbers in bold on the diagonal are the square roots of AVE; correlations are all significant at the 0.01 level **p < 0.01.

### 4.3 Dimensional heterogeneity comparison: analysis of similarities and differences in listening/reading vs. speaking/writing (test of H2)

Hypothesis H2 of this study presumed that because different communicative modalities are accompanied by varying degrees of face threat, the positive predictive strength of AI intelligent environment perception on “receptive willingness (listening/reading)” would be significantly stronger than that on “productive willingness (speaking/writing).” Data from the paired-samples *t*-test demonstrated that learners’ baseline willingness for receptive WTC (*M* = 3.66, *SD* = 0.71) was significantly higher than that for productive WTC (*M* = 3.10, *SD* = 0.72), with the difference reaching a statistically significant level, *t*(959) = 24.06, *p* < 0.001, Cohen’s *d* = 0.79. This indicates that in college English learning scenarios, productive tasks like speaking and writing still hold higher social evaluation risks and face threats, making learners’ foundational WTC distinctly lower than in receptive tasks like listening and reading.

**Figure 2:**
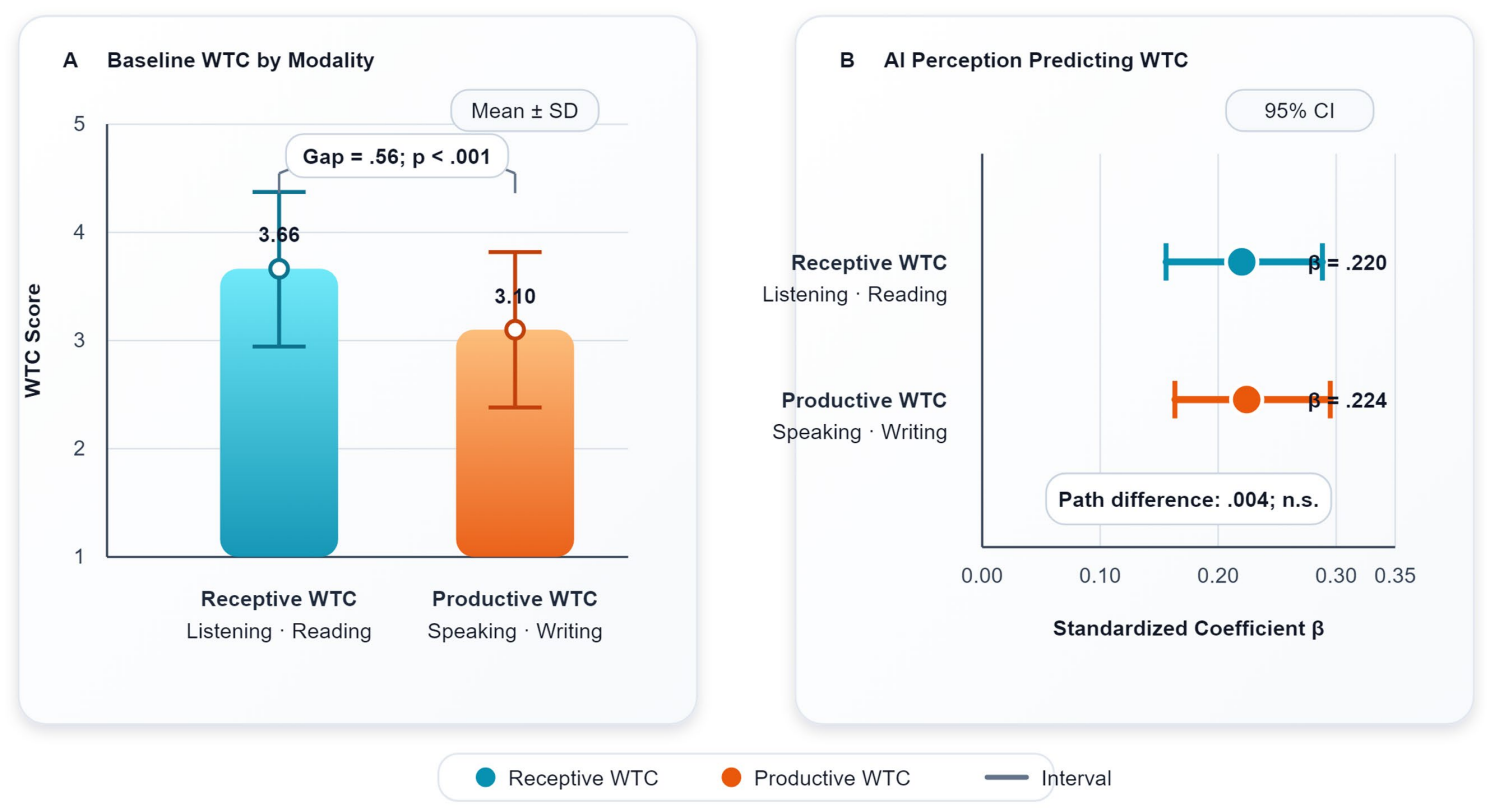
Foundational differences between receptive and productive L2 WTC and comparison of AI perception predictive strength. **(A)** The mean differences between Receptive WTC and Productive WTC, with error bars indicating standard deviations; **(B)** The standardized path coefficients and 95% confidence intervals from AI perception to both types of WTC. WTC, willingness to communicate; Receptive WTC, receptive willingness to communicate; Productive WTC, productive willingness to communicate; n.s., not significant.

However, comparing the path coefficients for receptive and productive WTC revealed a result contrary to the original hypothesis but offering substantial explanatory value: the predictive coefficients of AI perception for receptive WTC and productive WTC were nearly identical. Specifically, the standardized path coefficient from AI perception to receptive WTC was *β* = 0.220, 95% CI [0.156, 0.288]; to productive WTC, it was *β* = 0.224, 95% CI [0.163, 0.295]. The two sets of confidence intervals highly overlapped, and the path coefficient difference was extremely small, Δ*β* = 0.004, not reaching statistical significance. These key differences in foundational willingness and core predictive strength across multiple modalities are shown in Figure 2.

As seen in Figure 2, the foundational mean of receptive WTC is obviously higher than productive WTC. However, the predictive strength of AI perception on both types of WTC showed no statistically significant difference. This finding suggests that AI’s “de-socialized evaluation” interaction attribute not only helps with low-exposure-risk tasks like listening and reading but may also exert a psychological safety buffering effect in high-exposure-risk tasks like speaking and writing.

In summary, the validation results failed to support Hypothesis H2b (the differential path strength hypothesis). While the data substantiated the theoretical premise of Hypothesis H2a (that productive WTC exhibits a lower baseline mean), the positive predictive strengths of AI perception on the two communicative dimensions did not differ significantly. Consequently, the assertion that “AI assistance holds a stronger predictive power for receptive willingness” is not empirically supported, nor can we definitively claim statistical equivalence without an *a priori* equivalence bounds test (e.g., TOST). Nevertheless, this unanticipated outcome yields a highly valuable empirical discovery: it underscores that generative AI acts as a versatile “psychological safe haven,” equally empowering learners to overcome emotional barriers in high-risk productive tasks.

### 4.4 The mediating mechanism of self-efficacy (verification of H3)

To test the mechanism between perception and willingness, a Structural Equation Model (SEM) was established and a Bootstrap sampling was used to test the mediation effect. As shown in Table 4, after controlling for statistical variables, AI perception was significantly and positively correlated with self-efficacy (*β* = 0.346, *p* < 0.001). There is a significant correlation between the instant feedback brought by technological assistance and learners’ competence perception.

**Table 4:** Mediation effect bootstrap test report (5,000 samples).

Path relationship	Effect value (effect)	SE	95% CI (lower)	95% CI (upper)	Significance
Total effect (AI → WTC)	0.226	0.031	0.198	0.320	***
Direct effect (AI → WTC)	0.099	0.028	0.077	0.187	***
Indirect effect (AI → SE → WTC)	0.127	0.015	0.099	0.157	***

Bootstrap sampling was run 5,000 times; CI not containing 0 indicates a significant effect, ***p < 0.001. The overall model predictive power *R*^2^ = 0.231.

**Figure 3:**
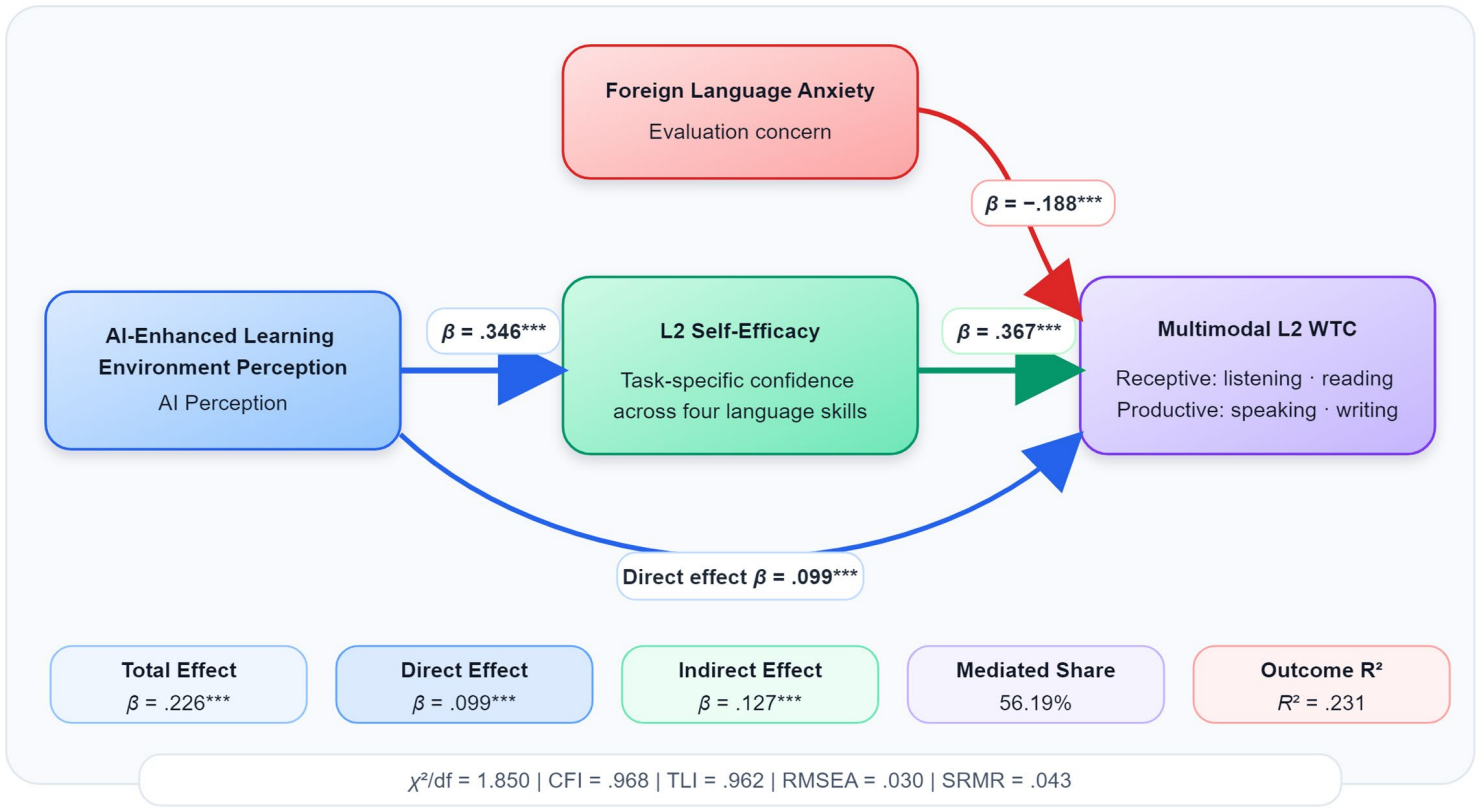
Structural path results of AI perception, self-efficacy, foreign language anxiety, and multimodal L2 willingness to communicate. Values in the figure represent standardized path coefficients. Solid arrows indicate significant paths, ***p < 0.001. FLA, foreign language learning anxiety; WTC, willingness to communicate.

This result supports Hypothesis H3: Self-efficacy plays a significant mediating role between AI perception and multimodal L2 WTC, with the indirect effect accounting for 56.19% of the total effect. To visually present this mediating path and relative effect decompositions, this study plotted the structural path diagram as shown in Figure 3.

### 4.5 Anxiety as a moderating variable and group heterogeneity expressions (verification of H4 and H5)

To rigorously test the moderating effect of Foreign Language Anxiety (FLA), namely Hypothesis H4, and simultaneously account for measurement errors inherent in the survey items, this study adopted the Product-Indicator Approach within the Structural Equation Modeling (SEM) framework (Ping, 1995). Specifically, the indicator variables of Self-Efficacy (SE) and FLA were mean-centered to prevent multicollinearity, after which their matched pairwise products were generated to serve as the indicators for the latent interaction construct (SE × FLA). The SEM estimation was conducted using Maximum Likelihood. The comprehensive moderation results are shown in Table 5.

**Table 5:** Moderating effect testing via latent interaction modeling (SEM).

Structural path	Unstandardized estimate (*B*)	Standard error (*SE*)	*z*-value	*p*-value
SE → Multimodal WTC	0.496	0.051	9.760	<0.001
FLA → Multimodal WTC	−0.191	0.042	−4.493	<0.001
Interaction (SE × FLA) → Multimodal WTC	0.129	0.071	1.823	0.068

*N* = 960. The estimators are unstandardized due to the mathematical scaling variations in the product-indicator approach.

As shown in Table 5, after correcting for measurement errors, both main effects remained highly significant. Self-efficacy has a robust positive predictive effect on multimodal L2 WTC (*B* = 0.496, *p* < 0.001), indicating that the stronger learners’ beliefs are in their L2 abilities, the higher their willingness to participate in multimodal communicative activities. The main effect of foreign language learning anxiety is significant and negative (*B* = −0.191, *p* < 0.001), signifying that higher anxiety levels are associated with lower communication willingness.

**Figure 4:**
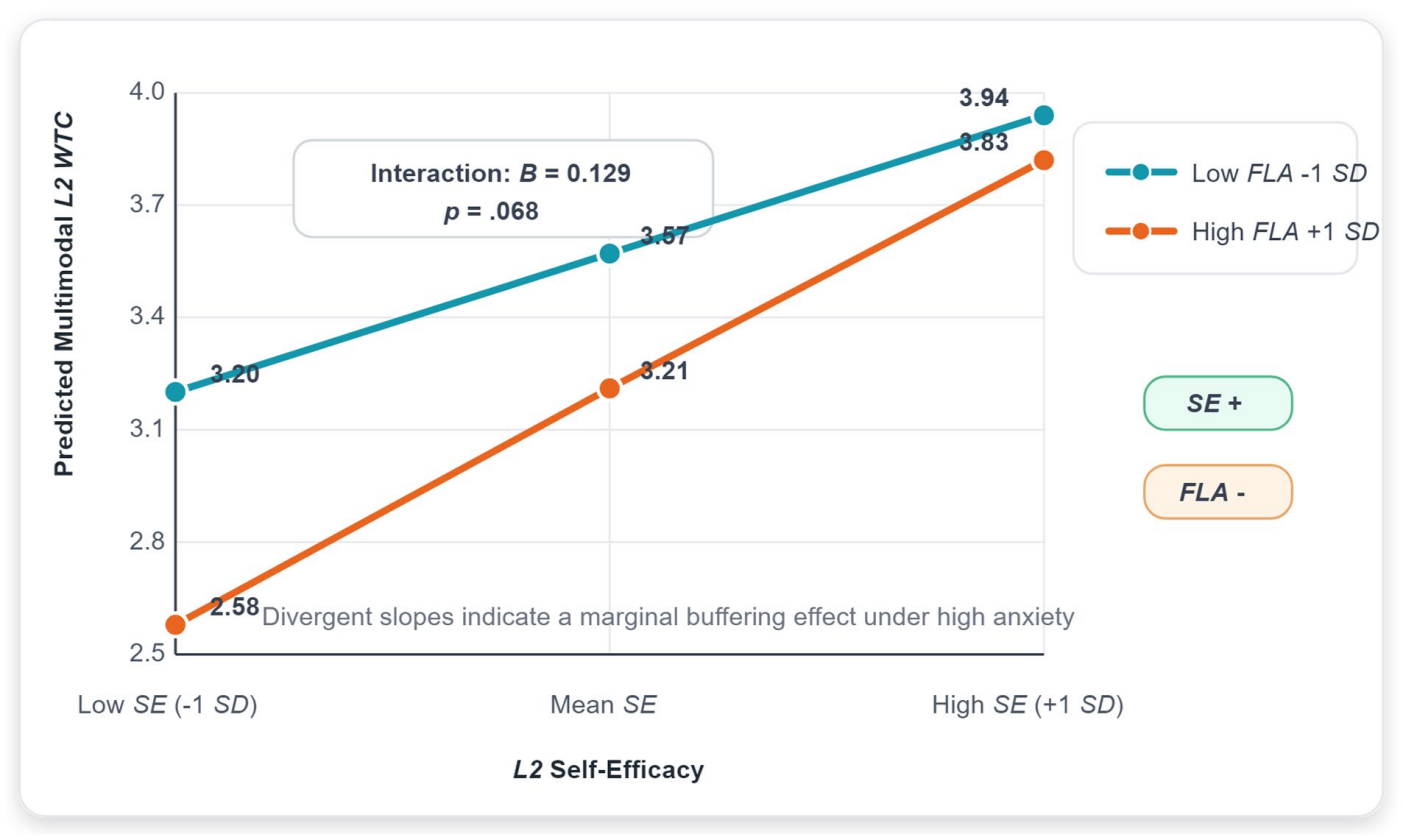
Simple slopes analysis of foreign language learning anxiety on the path between self-efficacy and multimodal L2 willingness to communicate. The figure illustrates the predicted morphological trend of the latent interaction based on the SEM model. Low FLA and High FLA represent foreign language learning anxiety 1 standard deviation below and above the mean, respectively. The divergence of the slopes indicates a buffering effect of self-efficacy under different anxiety conditions. FLA, foreign language learning anxiety; SE, L2 self-efficacy; WTC, willingness to communicate.

Importantly, the latent interaction term (SE × FLA) exhibited a positive coefficient on WTC (*B* = 0.129, *p* = 0.068, 95% CI [−0.010, 0.268]). Although it narrowly missed the strict 0.05 alpha level, it reached marginal significance (*p* < 0.10) and its 95% confidence interval slightly intersects with zero. The inclusion of the latent interaction term contributed an incremental variance explanation (Δ*R*^2^) of approximately 1.1% to multimodal WTC over the main-effects-only model. Consequently, because this moderation effect does not reach conventional significance levels (*p* < 0.05), it should be interpreted with caution. We present this pattern primarily as an exploratory finding that points to a potential emotional buffering tendency within technology-mediated contexts, a dynamic that warrants further empirical replication. To further display the morphology of the latent moderating effect, this study plotted a simple slopes map, as shown in Figure 4.

As illustrated in Figure 4, simple slopes analysis was projected at ±1 SD around the mean of FLA. The positive interaction coefficient (*B* = 0.129) suggests a buffering effect. Specifically, although overall WTC stays lower for students with high learning anxiety, a robust sense of self-efficacy remains highly capable of buffering this anxiety-induced silence, boosting their willingness to communicate at a dynamically steeper rate compared to their low-anxiety peers. In other words, in intelligent learning environments, while anxiety holds a significant negative association with willingness to communicate, a high level of self-efficacy effectively compensates for this negative impact, stimulating communication engagement even under high-anxiety conditions. This finding aligns with the “de-socialized evaluation” and “low-pressure trial-and-error” traits of AI-supported learning environments, where learners feel safer leveraging their self-efficacy to overcome emotional barriers.

In conclusion, Hypothesis H4 is partially supported. The results reveal that rather than simply exerting a rigidly negative moderation, foreign language learning anxiety acts as an emotional stressor whose negative impact can be marginally yet effectively buffered by strong self-efficacy. This indicates that within intelligent learning contexts, the interplay between cognitive beliefs (SE) and emotional factors (FLA) forms a compensatory mechanism rather than absolute mutual inhibition (Table 6).

**Table 6:** Measurement invariance test across English proficiency groups.

Model	*χ* ^2^	*df*	*χ*^2^/*df*	CFI	RMSEA	ΔCFI
Configural invariance	184.32	104	1.77	0.998	0.021	—
Metric invariance	198.45	128	1.55	0.998	0.017	<0.001

ΔCFI <0.01 is the threshold for assuming metric invariance across groups.

Furthermore, to examine the potential group heterogeneity across English proficiency levels (H5), this study conducted a structured multi-group SEM analysis. Before comparing the structural path coefficients, measurement invariance was examined through two progressive steps: configural invariance and metric invariance. The configural model, which allowed factor loadings to be freely estimated across groups, yielded an excellent fit (*χ*^2^/*df* <2.50, CFI = 0.998, RMSEA = 0.021). The subsequent metric invariance model, where factor loadings were constrained to be equal across the four English level groups, also demonstrated high stability (ΔCFI < 0.001). This result signifies that the core latent constructs were measured in a comparable manner across different proficiency levels, fulfilling the prerequisite for cross-group path comparisons.

After establishing measurement invariance, the structural path coefficients of “AI Perception → Self-Efficacy” were estimated for each proficiency group. As presented in Table 7, the positive path association from AI perception to self-efficacy remained statistically significant across all proficiency groups, with Critical Ratios (*z*-values) well exceeding the threshold of 1.96 (*p* < 0.01).

**Table 7:** Comparison of path coefficients and critical ratios among English level groups.

English level group	Sample size (*N*)	Path estimate (*B*)	S.E.	C.R. (*z*-value)	*p* value
Group 1: beginner (below CET-4)	144	0.312	0.090	3.467	0.001
Group 2: CET-4 level	413	0.479	0.078	6.141	<0.001
Group 3: CET-6 level	303	0.326	0.066	4.939	<0.001
Group 4: high cert. level	100	0.326	0.106	3.075	0.002

B represents the unstandardized path coefficient from AI perception to self-efficacy; S.E., standard error; C.R., critical ratio; significant at |C.R.| > 1.96.

Because specific structural path constraint tests (i.e., chi-square difference tests for constrained vs. unconstrained models) were not performed, we refrain from claiming magnitude differences across proficiency levels. Instead, the consistent manifestation of positive and significant paths across all groups—from beginners to advanced learners—suggests that the proposed theoretical framework maintains reasonable cross-group stability. In this sense, H5 received preliminary empirical support regarding the model’s generalizability rather than comparative differentiation.

### 4.6 Triangulation analysis from a qualitative perspective

To provide textual triangulation for the quantitative data, this study employed a thematic analysis method to encode and summarize the open-ended data as shown in Table 8. Regarding the process of AI-assisted foreign language learning, subjects mainly focused on “Functional support and efficiency.” Among these, “Emotional safety (low pressure),” mentioned 165 times, offered a possible qualitative interpretation for the exploratory buffering tendency observed in the quantitative model: “Because there’s no need to worry about real human social judgments, dialoguing with AI vastly lowers the cost of trial and error.” Additionally, the qualitative materials highlighted “Cognitive/dependency risks” with up to 180 occurrences and “Lack of real interaction” with 81 occurrences. These raw narratives echo the “dark side” of technological integration discussed by Honggang et al. (2026) and Khoso et al. (2025c). They point out that human-computer interaction ultimately cannot wholly replace authentic human communication with its accompanying heart-rate spikes in social settings; over-reliance on generative algorithms potentially triggers digital fatigue and erodes individuals’ autonomous communicative resilience once detached from screens. This finding supplies an important footnote for the rational boundaries of technology, emphasizing a balanced perspective on AI-mediated EFL environments.

**Table 8:** Thematic mapping analysis of open-ended feedback.

Theme	Frequency	Representative quote
Functional support and efficiency	241	“AI helped tremendously in academic English reading, summarizing literature structures for me, greatly easing reading pressure.”
Emotional safety (low pressure)	165	“Facing an AI language partner, I can boldly try and make mistakes without worrying about being judged or mocked for being tongue-tied, unlike speaking with a foreign teacher.”
Cognitive/dependency risks	180	“It sure is fast to one-click translate complex sentences, but I feel myself increasingly dependent on it. I’m actually more confused when it comes to independent thinking.”
Lack of real interaction	81	“Lacks the contextual pressure of real scenarios. That kind of tension when talking to a real human is something machines cannot simulate; I’d still get stage fright running into foreigners.”

## 5 Discussion

### 5.1 Correlation characteristics between intelligent environment perception and multimodal WTC

The current data indicate that intelligent environment perception shares a significant positive association with multimodal L2 WTC (Deng et al., 2024). When algorithms provide prompt grammar corrections and contextual translations, their role as cognitive scaffolding correlates directly with the release of psychological resources for learners engaging in communication.

Reflecting on the modality heterogeneity (Hypothesis H2), the findings offer an unexpected yet insightful pattern. Descriptive data corroborated Hypothesis H2a, confirming that learners exhibit a significantly lower baseline WTC for productive tasks compared to receptive tasks, aligning with the premise that speaking and writing inherently carry higher systemic face threats. However, the path analysis results did not support Hypothesis H2b, as there was no statistically significant difference in the predictive strength of AI perception on receptive willingness (*β* = 0.220) versus productive willingness (*β* = 0.224). This absence of a significant difference challenges traditional assumptions that “tech support exhibits stronger positive associations predominantly with low-pressure receptive tasks.” Instead, it suggests that generative AI’s “de-socialized evaluation” attribute functions broadly as a “psychological safe haven,” equally and robustly supporting learners to confront high-risk language production tasks.

### 5.2 The mediating role of L2 self-efficacy

The quantitative analysis indicated that self-efficacy accounts for 56.19% of the indirect association between AI perception and willingness to communicate. This pattern aligns with Bandura’s (1997) competence belief theory, suggesting that the utility of external tools correlates with an individual’s internal sense of capability, which in turn aligns with substantive behavioral intentions.

In AI-assisted contexts, this specific efficacy link is noteworthy. When learners utilize AI to receive accurate feedback—such as fixing expression errors or optimizing sentence structures—they can accumulate domain-relevant confidence via high-frequency vicarious success experiences. Theoretically, a distinction must be drawn between “Proxy Agency” and “Self-Efficacy”: if learners purely attribute task accomplishments to algorithmic power, their communication willingness might collapse once detached from technology. However, the observed mediating pattern suggests an alternative—that appropriate intelligent assistance correlates with learners internalizing success experiences into affirmations of their own abilities. However, acknowledging the “dark side” of technological integration is crucial here. If learners continually bypass independent cognitive effort in favor of instant AI processing, they risk falling into digital fatigue and AI dependency, which ultimately restricts sustainable vocabulary acquisition and communicative resilience (Khoso et al., 2025c; Honggang et al., 2026). Therefore, the mediating role of self-efficacy is not merely a statistical bridge but a critical cognitive threshold: it is only when AI perception converts into internalized confidence that learners can avoid proxy agency and achieve genuine communicative readiness.

### 5.3 The moderating effect of anxiety and group differences

A primary finding concerns the dual role of Foreign Language Anxiety (FLA) in intelligent learning environments. Looking at the main associations, the latent SEM results show that FLA was significantly and negatively associated with multimodal WTC. This suggests that, even in AI-supported learning contexts, learners with higher anxiety levels still tend to report lower baseline willingness to communicate. The relatively low-pressure nature of human-computer interaction does not appear to eliminate anxiety-related differences in communicative willingness.

Despite this persistent baseline anxiety, the latent interaction between self-efficacy and FLA reveals a more nuanced association pattern. The interaction term between SE and FLA was positive and marginally significant, suggesting a potential buffering tendency. Specifically, although high-anxiety learners generally showed lower levels of WTC, the positive association between self-efficacy and WTC appeared to be relatively stronger under higher anxiety conditions. This pattern indicates that anxiety did not simply function as a uniformly negative boundary condition in the relationship between self-efficacy and WTC. Rather, the data are consistent with a compensatory association in which stronger self-efficacy corresponds to higher communicative willingness even among learners reporting elevated anxiety.

This result is compatible with the “desensitization attributes” and “de-socialized evaluation” features of AI-supported learning environments. Compared with face-to-face interaction, human-computer interaction may involve fewer immediate social-evaluative cues from human interlocutors. Therefore, learners’ concerns about errors and loss of face may be less salient in such contexts. Within this relatively low-pressure environment, localized successful experiences with AI feedback may be associated with stronger self-efficacy beliefs, which in turn correspond to higher WTC even among anxious learners. Thus, the current findings point to a correlational pattern between cognitive belief and emotional pressure: anxiety is associated with a lower baseline level of WTC, whereas self-efficacy is associated with sustained communicative willingness across different anxiety levels.

Adding to this psychological profile, group comparisons based on baseline English levels (H5) yielded theoretically meaningful exploratory findings. The path coefficients between AI perception and self-efficacy remained consistently positive and statistically significant across all proficiency levels—from beginners to advanced credential holders. Because rigorous chi-square difference constraints were not applied, we avoid claiming comparative magnitude differences among these clusters. Instead, the persistent significance of these associative paths demonstrates the robustness and cross-group stability of the proposed structural model. This finding implies that AI environments can universally benefit learners’ competence beliefs, bridging the gap between “wanting to express” and “not knowing how to express accurately” across varying stages of language acquisition.

## 6 Conclusion and prospects

### 6.1 Research summary

Through a cross-sectional mixed-methods study involving 960 college students, this research elucidated the structural relationships surrounding L2 willingness to communicate (WTC) within intelligent learning environments. Survey data indicate that learners’ perception of AI not only positively correlates with their overall WTC and exerts comparable predictive strength across both receptive (listening/reading) and productive (speaking/writing) modalities. Within this structural path, L2 self-efficacy served as an important indirect link. The latent moderation analysis further revealed that foreign language anxiety was significantly and negatively associated with multimodal WTC. Importantly, the interaction between self-efficacy and anxiety presented a marginally significant buffering tendency (*p* = 0.068), functioning as an exploratory pattern requiring future validation. This suggests that although higher anxiety corresponds to lower baseline WTC, stronger self-efficacy appears capable of stimulating relatively higher communicative engagement even under stressful conditions. Subgroup comparisons indicated consistent model stability across all proficiency levels, from beginners to advanced users. Supporting these structural patterns, qualitative feedback corroborated the quantitative data, highlighting AI’s role as a “psychological safety buffer” while also delineating the practical boundaries between virtual AI assistance and authentic interpersonal communication.

### 6.2 Teaching implications

These cross-sectional findings offer practical recommendations for foreign language instruction in the digital phase. To operationalize these results, we propose a “Scaffolded AI-WTC Integration Model.”

Firstly, given that AI’s correlation strengths hold comparable in both listening/reading and speaking/writing modalities, teachers may integrate generative AI as scaffolding in writing and oral production courses, by designing “low-consequence” interaction zones. For instance, through tasks like “AI-Mediated Peer Dialogue,” students can co-construct dialogs with AI simulators to seek initial feedback and refine their expressions before interacting with human peers. Secondly, considering the observed buffering tendency associated with self-efficacy under anxiety-related conditions, instructional design should attend not only to anxiety reduction but also to the cultivation of perceived communicative competence. Teachers may create staged AI-supported tasks in which learners first receive low-pressure feedback from intelligent systems and then gradually transfer revised expressions into peer interaction, classroom presentation, or authentic interpersonal communication.

Ultimately, the focus of educational interventions should gradually transition from “instructing students to utilize tools” to “forging cognitive confidence through tools.” Teachers need to design reasonable transitional “scaffolding” tasks shifting from human-machine dialogs to interpersonal dialogs, thereby preventing students from stalling at the “proxy agency” echelon, guiding them to decouple from technology to independently accomplish communication post-obtaining AI feedback. By achieving this progressive return from tool dependencies back to autonomous communicative subjects, learners—particularly intermediate groupings like CET-4 students—can benefit from more targeted AI-based error correction and reconstruction training architectures slanted toward their specific proficiency needs to heighten intervention benefits.

### 6.3 Limitations and research prospects

This study contains certain limitations that demand gradual refining in subsequent explorations.

Regarding the psychometric modeling and data sources, the current study relied entirely on self-reported survey data. First, although Harman’s Single-Factor Test indicated acceptable parameters, it is widely acknowledged as an insensitive diagnostic for common method bias (CMB) (Podsakoff et al., 2012). Future studies should incorporate multi-source or objective behavioral data to parse out potential method variance comprehensively. Second, in the CFA model, some item residuals were correlated based on Modification Indices. While theoretically justified within the same latent constructs (Byrne, 2016), this hints that certain scale items possess semantic overlap. Future research needs to refine measurement tools to boost intrinsic model parsimony without relying on post-hoc modifications.

Methodologically, the cross-sectional research design mainly discloses the correlational relationship among variables, possessing limited causative inferential power for psychological evolutions. Future studies could employ longitudinal studies or experimental intervention designs to accurately measure the dynamic evolutionary trajectories of students’ communicative willingness 3–6 months before and after AI tool implementations; concurrently, seeing that a reciprocal dynamic enhancement relationship rather than one-way conduction might exist among “perception—efficacy—willingness,” longitudinal designs would also help further clarify the temporal causality among the trio, thereby rendering a more stringent causal inference foundation for the mediation paths unveiled by this study. Moreover, although the proposed model identified meaningful associations among AI perception, self-efficacy, anxiety, and multimodal WTC, its overall predictive power for WTC remained moderate (*R*^2^ = 0.231). This indicates that a substantial proportion of variance in learners’ communicative willingness remains unexplained. Future research should incorporate additional individual, motivational, instructional, and behavioral predictors, such as personality traits, L2 motivation, classroom climate, teacher support, actual AI-use frequency, and objective communicative performance, to provide a more comprehensive explanatory framework for multimodal L2 WTC. Likewise, the marginally significant latent interaction between self-efficacy and foreign language anxiety should be interpreted as an exploratory associational tendency rather than causal evidence, and its stability requires further verification across different learning contexts and proficiency groups.

From a behavioral perspective, the qualitative materials presented the risk of “cognitive laziness under machine dependency,” so future psychological research should further probe the changing trends of learners’ cognitive autonomy over prolonged human-machine interactions, so as to supply more comprehensive scientific bases for AI educational ethics and application thresholds.

Finally, as this study was conducted within a single university in Northwest China, the generalizability of the results may be subject to specific institutional and cultural nuances, such as Chinese students’ unique “face concern” and social communication norms. Future research should cover more diverse geographical locations and institutional types to validate these findings.

## Data Availability

The original contributions presented in the study are included in the article/Supplementary material, further inquiries can be directed to the corresponding author.
