# Supplementary material for: A study on the correlation between the perception of intelligent college English learning environments and the willingness to communicate in listening, speaking, reading, and writing
Source: Front Psychol. 2026 Jul 8;17:1885211. doi: 10.3389/fpsyg.2026.1885211 (PMC13388837; doi:10.3389/fpsyg.2026.1885211)
Supplement: Supplementary file 1 [file Data_Sheet_1.ZIP › Supplementary_Material_1_Bilingual_Questionnaire.docx]

# Supplementary Material

**Appendix A: The Bilingual Survey Instrument (中英文双语对照问卷工具)**

**Note:** The survey was originally administered in Chinese to the participants. The English translations were produced using a translation and back-translation procedure to ensure conceptual equivalence. All quantitative matrix items were rated on a 5-point Likert scale (1 = Strongly Disagree, 2 = Disagree, 3 = Neutral/Uncertain, 4 = Agree, 5 = Strongly Agree).

### Part 1: Informed Consent & Demographics (知情同意与人口学特征)

| Variable Code | Original Chinese Item (原始中文) | English Translation (英文翻译) | Scale / Options |
| --- | --- | --- | --- |
| Consent | （知情同意）我已阅读上述信息，并自愿参与本次匿名调查。 | I have read the above information and voluntarily participate in this anonymous survey. | 1=Yes; 0=No (Terminate) |
| Gender | 您的性别是： | What is your gender? | 1=Male (男); 2=Female (女) |
| Grade | 您的年级是： | What is your academic year? | 1=Freshman; 2=Sophomore; 3=Junior; 4=Senior or above |
| Major | 您目前所在的专业大类属于： | What is your major category? | 1=STEM; 2=Business/Social Sci; 3=Humanities/Edu; 4=Arts/Sports/Other |
| Eng_Level | 您当前的英语水平比较符合以下哪一项？ | Which of the following best describes your current English proficiency? | 1=None; 2=CET-4; 3=CET-6; 4=Advanced Certifications (IELTS/TOEFL) |

### Part 2: Quantitative Scales (定量量表测项)

| Construct (构念) | Code | Original Chinese Item (原始中文测项) | English Translation (英文翻译) |
| --- | --- | --- | --- |
| AI Perception *(AI智能感知)* | AI_1 | 借助 AI 辅助工具极大地减轻了我在完成英语客观任务时的思维负担。 | AI significantly reduces my cognitive load when completing objective English tasks. |
|  | AI_2 | 这些智能化工具能够针对我的英语薄弱环节提供即时且有逻辑的反馈。 | Smart tools provide instant and logical feedback targeting my specific English weaknesses. |
|  | AI_3 | 使用智能工具提升了我处理长篇外文材料时的效率与愉悦感。 | Using AI increases the efficiency and enjoyment of processing long foreign texts. |
|  | AI_4 | 对我而言，随时利用移动端的智能设备进行英语查漏补缺非常便捷。 | It is highly convenient for me to use mobile smart devices for fragmented English learning. |
|  | AI_5 | 当英语学习遭遇困难时，我倾向优先向 AI 工具提问，而非直接跳过。 | When facing difficulties, I tend to consult AI tools first rather than skipping the problem. |
| L2 Self-Efficacy *(自我效能感)* | SE_1 | 即使语速偏快，我也有信心能捕捉到纯英语听力材料中的核心信息。 | I am confident in catching the core information in fast-paced spoken English materials. |
|  | SE_2 | 我相信凭借自己的积累，能够顺利读懂与我专业相关的英文原典或学术报道。 | I believe I can successfully read academic literature and original texts related to my major. |
|  | SE_3 | 当置身于需要用英语交谈的真实环境时，我相信自己能把基本需求表达清楚。 | I am confident in expressing my basic needs clearly in real-life English communication settings. |
|  | SE_4 | 我相信自己有能力运用恰当的词汇和语法，写出一篇条理清晰的英文小短文。 | I am confident in writing a well-structured short English essay using appropriate grammar. |
| Foreign Language Anxiety *(外语焦虑)* | FLA_1 | 当我认为周围同学的英语水平都比我高时，我会感到很有压力。 | I feel pressured when I think my peers have higher English proficiency than I do. |
|  | FLA_2 | 在没有提前准备的情况下被要求使用英语进行回应，会让我感到恐慌。 | I feel panicked when asked to respond in English without advance preparation. |
|  | FLA_3 | 哪怕知道犯错是很正常的，我内心仍然会害怕别人嘲笑我的英文发音或语病。 | Even knowing mistakes are normal, I still fear others mocking my pronunciation or grammar. |
|  | FLA_4 | 在面对重要的英语技能评估或输出任务前，我往往会感到焦虑和不安。 | I often feel anxious and uneasy before important English assessments or productive tasks. |
| Receptive WTC *(听读交际意愿)* | WTC_R_1 | [听] 若网络平台推送了一段无字幕的原版英语演讲或访谈视频，我愿意耐心看完听完。 | [Listening] I am willing to patiently listen to an uncaptioned original English video block. |
|  | WTC_R_2 | [听] 公共场合若有外国友人用英语交谈，我愿意在旁留心倾听以锻炼听感。 | [Listening] I am willing to pay attention to foreigners conversing in English in public spaces. |
|  | WTC_R_3 | [读] 当国外网站弹出一条我感兴趣的全英文资讯界面，我愿意去阅读并尝试理解。 | [Reading] I am willing to try comprehending a full English web page that catches my interest. |
|  | WTC_R_4 | [读] 为拓宽视野，我愿意主动搜集并研读未经翻译的海外原版文本材料。 | [Reading] To broaden my horizons, I am willing to read untranslated foreign text materials. |
| Productive WTC *(说写交际意愿)* | WTC_P_1 | [说] 在课堂或分享会上，当有机会用英语发表观点时，我愿意主动争取发言。 | [Speaking] I am willing to actively seek opportunities to express my opinions in English classes. |
|  | WTC_P_2 | [说] 在校园内外若偶遇需要帮助的国外游客或留学生，我愿意主动上前用英语搭话。 | [Speaking] I am willing to step forward and initiate a conversation to help a lost foreigner. |
|  | WTC_P_3 | [写] 我愿意尝试在社交平台上，偶尔坚持用英文记录心情短语。 | [Writing] I am willing to occasionally write social media lifestyle updates in English. |
|  | WTC_P_4 | [写] 如果有机会结识海外虚拟语伴或笔友，我愿意花费精力用英文发送长段落消息。 | [Writing] I am willing to spend effort composing long English messages to overseas pen pals. |

### Part 3: Qualitative Open-ended Questions (质性开放问题) - *Optional (选填)*

| Variable Code | Original Chinese Prompt (原始中文提问) | English Translation (英文翻译) | Format |
| --- | --- | --- | --- |
| Qual_Q1 | 请简要回忆并描述一次：某款 AI 工具让您觉得“真的有帮到我缓解压力”或相反“让我产生了依赖/迷茫”的情境。当时您最真实的心理感受是怎样的？ | Please describe a specific situation where an AI tool either helped relieve your English learning pressure or caused significant dependency/confusion. What were your authentic psychological feelings? | String (Text box) |
| Qual_Q2 | 相较于面对传统课堂里的真人老师，您在面对机器 AI 进行英语互动时，心理上最明显的变化是什么？（例如：更放松不怕犯错、觉得冰冷没有感情等） | Compared to facing a human teacher, what is the most prominent psychological change when interacting with an AI tool for English? (e.g., feeling more relaxed to make mistakes, feeling it lacks warmth, etc.) | String (Text box) |
