# Supplementary material for: A study on the correlation between the perception of intelligent college English learning environments and the willingness to communicate in listening, speaking, reading, and writing
Source: Front Psychol. 2026 Jul 8;17:1885211. doi: 10.3389/fpsyg.2026.1885211 (PMC13388837; doi:10.3389/fpsyg.2026.1885211)
Supplement: Supplementary file 1 [file Data_Sheet_1.ZIP › Supplementary_Material_3_SEM_Modification_Report.docx]

### **Supplementary Material 3: SEM Model Modification Report**

Following the guidelines of Byrne (2016) and Kline (2023), model modifications were conducted to account for measurement redundancies. To avoid over-fitting, residual correlations were strictly limited to items within the same latent construct with Modification Indices (MI) > 10.0. No cross-factor correlations were established.

**Table S1. Comparison of Model Fit Indices (Before and After Modification)**
The initial model exhibited an acceptable fit. The introduction of three intra-factor residual correlations improved the fit indices to the optimal level reported in Section 4.1.

| Model Status | χ² | df | χ²/df | CFI | TLI | RMSEA | SRMR |
| --- | --- | --- | --- | --- | --- | --- | --- |
| Initial Model | 456.78 | 186 | 2.456 | 0.932 | 0.918 | 0.054 | 0.049 |
| Modified Model | 338.55 | 183 | 1.850 | 0.968 | 0.962 | 0.030 | 0.043 |

**Table S2. List of Established Residual Correlations and Theoretical Rationale**
The three modified residual pairs, their respective MI values, and theoretical justifications are listed below.

| Residual Pair | MI Value | Theoretical Justification |
| --- | --- | --- |
| **e1 (AI_1) ↔ e2 (AI_2)** | 22.45 | **Semantic Overlap:** Both items assess the immediate “efficiency” and “correctness” of AI tools. Their high semantic similarity regarding technological speed leads to shared measurement variance. |
| **e8 (SE_3) ↔ e9 (SE_4)** | 18.12 | **Modality Homogeneity:** Both items measure self-efficacy in “productive” tasks (speaking and writing). Output-based language tasks require similar cognitive efforts, causing localized response dependence. |
| **e15 (WTC_R_1) ↔ e16 (WTC_R_2)** | 14.33 | **Contextual Feature:** Both items examine willingness in “receptive” modes (listening and reading). The passive nature of information decoding naturally elicits similar psychological responses. |
